# The impact of portal vein tumor thrombosis on survival in patients with hepatocellular carcinoma treated with different therapies: A cohort study

**DOI:** 10.1371/journal.pone.0249426

**Published:** 2021-05-07

**Authors:** Aline Mähringer-Kunz, Verena Steinle, Roman Kloeckner, Sebastian Schotten, Felix Hahn, Irene Schmidtmann, Jan Bernd Hinrichs, Christoph Düber, Peter Robert Galle, Hauke Lang, Arndt Weinmann

**Affiliations:** 1 Department of Diagnostic and Interventional Radiology, University Medical Center Mainz, Mainz, Germany; 2 Department of Diagnostic and Interventional Radiology, University Hospital Heidelberg, Heidelberg, Germany; 3 Department of Internal Medicine I, University Medical Center Mainz, Mainz, Germany; 4 Institute for Diagnostic and Interventional Radiology and Neuroradiology, HSK Wiesbaden, Germany; 5 Department of Medical Biostatistics, Epidemiology and Informatics, Johannes Gutenberg University Mainz, Mainz, Germany; 6 Department of Radiology, Medical School Hannover, Hannover, Germany; 7 Department of General, Visceral and Transplant Surgery, University Medical Center Mainz, Mainz, Germany; 8 Clinical Registry Unit (CRU), University Medical Center Mainz, Mainz, Germany; Cincinnati Children’s Hospital Medical Center, UNITED STATES

## Abstract

**Background:**

Portal vein tumor thrombosis (PVTT) is a frequent complication of hepatocellular carcinoma (HCC), which leads to classification as advanced stage disease (regardless of the degree of PVTT) according to the Barcelona Clinic Liver Cancer Classification. For such patients, systemic therapy is the standard of care. However, in clinical reality, many patients with PVTT undergo different treatments, such as resection, transarterial chemoembolization (TACE), selective internal radiation therapy (SIRT), or best supportive care (BSC). Here we examined whether patients benefited from such alternative therapies, according to the extent of PVTT.

**Methods:**

This analysis included therapy-naïve patients with HCC and PVTT treated between January 2005 and December 2016. PVTT was classified according to the Liver Cancer study group of Japan as follows: Vp1 = segmental PV invasion; Vp2 = right anterior or posterior PV; Vp3 = right or left PV; Vp4 = main trunk. Overall survival (OS) was analyzed for each treatment subgroup considering the extent of PVTT. We performed Cox regression analysis with adjustment for possible confounders. To further attenuate selection bias, we applied propensity score weighting using the inverse probability of treatment weights.

**Results:**

A total of 278 treatment-naïve patients with HCC and PVTT were included for analysis. The median observed OS in months for each treatment modality (resection, TACE/SIRT, sorafenib, BSC, respectively) was 32.4, 8.1, N/A, and 1.7 for Vp1; 10.7, 6.9, 5.5, and 1.2 for Vp2; 6.6, 7.5, 2.9, and 0.6 for Vp3; and 8.0, 3.6, 5.3, and 0.7 for Vp4. Thus, the median OS in the resection group in case of segmental PVTT (Vp1) was significantly longer compared to any other treatment group (all *p* values <0.01).

**Conclusions:**

Treatment strategy for HCC with PVTT should not be limited to systemic therapy in general. The extent of PVTT should be considered when deciding on treatment alternatives. In patients with segmental PVTT (Vp1), resection should be evaluated.

## Introduction

Hepatocellular carcinoma (HCC) is a major global health problem [1], which is commonly complicated by portal venous tumor thrombosis (PVTT) [2,3]. The clinical relevance of PVTT is remarkable because of both its high incidence in patients with HCC (10–40%) [2,3] and its massive impairment of prognosis [4]. PVTT is a criterion for classifying HCC as advanced stage according to the widely accepted Barcelona Clinic Liver Cancer Classification (BCLC) [5], which is endorsed by the European Association for the Study of the Liver (EASL) [6] and the American Association of the Study of Liver Diseases (AASLD) [7].

The BCLC recommends palliative systemic therapy for patients with advanced stage disease, regardless of the extent of PVTT [8]. Despite the approval of new agents, the standard of care for patients with advanced stage HCC is still sorafenib, which is proven to be safe and effective in this patient subgroup [9,10]. However, in clinical reality, a high proportion of patients with PVTT are not treated strictly according to the BCLC algorithm but rather undergo individualized therapies, including resection, transarterial chemoembolization (TACE), selective internal radiation therapy (SIRT), or best supportive care (BSC) [11,12]. Moreover, several studies from Asia indicate that well-selected patients might benefit from such alternative therapies [13–15]. However, there is no consensus regarding how to identify these patients, and the level of evidence supporting such alternative treatments is low, especially among Western patients.

In the present study, we aimed to identify patients who might benefit from surgical or locoregional therapies, considering the extent of PVTT.

## Material and methods

### Study design, data acquisition, and patient recruitment

We performed a registry-based cohort study, and followed the STROBE guidelines when writing our manuscript (S1 Checklist) [16]. The need for institutional review board approval was waived by the Ethics Committee of the Medical Association of Rhineland Palatinate, Mainz, Germany. Informed consent was not applicable due to the retrospective nature of this cohort study. Patient records and information were anonymized prior to analysis.

We collected data regarding etiology of liver disease, baseline patient characteristics, tumor characteristics, and treatment from a prospectively populated clinical database that was installed in 1998 at our university medical center [17]. Laboratory results from the time of the initial HCC diagnosis were extracted from our laboratory information system. If no test results were available for the exact date of diagnosis, we selected results from the date nearest to the date of diagnosis if the interval was less than 90 days, otherwise the results were recorded as missing. For patients who were initially diagnosed outside of the university hospital, physician reports were requested, and the documented laboratory data were incorporated into our database.

Survival data were extracted from clinical records and by contacting registration offices. The recruitment period was January 1, 2005 to December 31, 2016. To ensure one year of follow-up, the final evaluation date was set as December 31, 2017. Inclusion criteria were HCC diagnosis according to EASL [6]/ AASLD [7], and PVTT diagnosis. Furthermore, patients undergoing combination therapy were not included into the analysis. Patients who received therapy for HCC prior to PVTT diagnosis were excluded. The presented methodology was partly described in a previous project investigating the entire cohort of patients with HCC in order to determine the impact of PVTT on overall survival (OS) [4].

### Imaging analysis

All imaging studies of the identified patients were semi-automatically requested. Subsequently, all analyzable patient images were transferred to a reserved and secured partition of our picture archiving and communication system (SECTRA, Linköping, Sweden). We identified all individual cross-sectional imaging examinations suitable for final analysis. Due to considerable heterogeneity of the labeling and the great variety of treatments performed, this selection process had to be performed manually. Then the imaging studies and the respective contrast-phases were arranged in the PACS viewer using predetermined layout rules.

**Fig 1:**
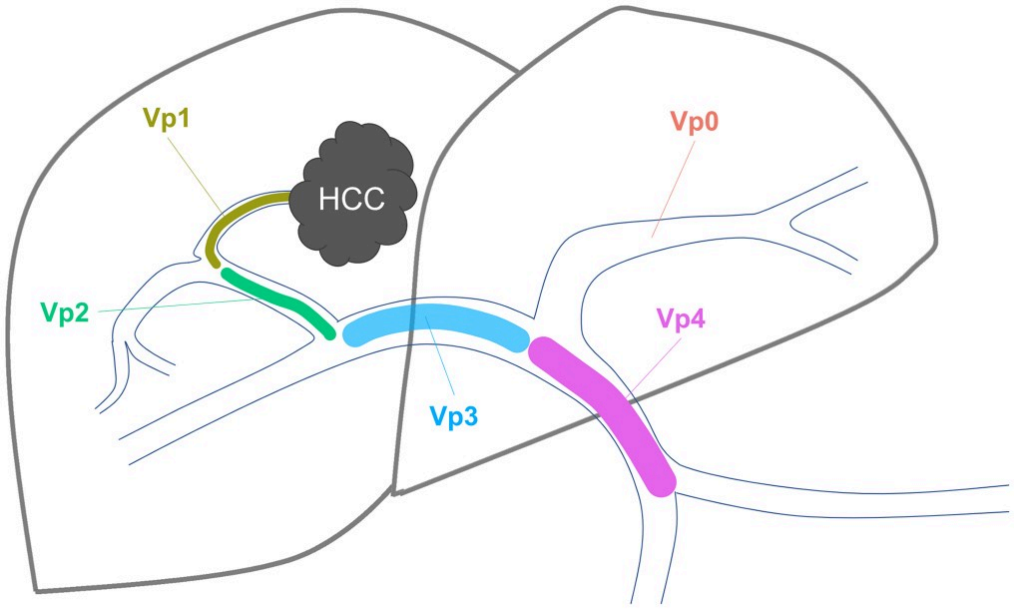
Figure describing the anatomical classification of portal vein tumor thrombosis as suggested by the Liver Cancer Study Group of Japan (LCSGJ). HCC, Hepatocellular carcinoma; Vp0 = no PVTT; Vp1 = segmental PV invasion; Vp2 = right anterior or posterior PV; Vp3 = right or left PV; and Vp4 = main trunk and/or contra-lateral portal vein branch to the primarily involved lobe.

Imaging analysis was performed through consensus by two board-certified radiologists with >10 years of experience in oncologic imaging of the liver (RK and SS). PVTT was retrospectively diagnosed by analyzing all available contrast-enhanced computed tomography (CT) or magnetic resonance imaging (MRI) scans. Bland thrombus and PVTT were differentiated using established criteria for CT and MRI [18]. In case of disagreement, both observers performed a joint second review to achieve a consensual decision. The extent of PVTT was documented in the HCC registry using the classification suggested by the Liver Cancer Study Group of Japan (LCSGJ) ranging from Vp0–Vp4, where Vp0 = no PVTT; Vp1 = segmental PV invasion; Vp2 = right anterior or posterior PV; Vp3 = right or left PV; and Vp4 = main trunk and/or contra-lateral portal vein branch to the primarily involved lobe [3,19], Fig 1.

### Treatment

Our interdisciplinary tumor board, including members from the departments of Hepatology, Diagnostic and Interventional Radiology, Hepatobiliary/Transplant Surgery, and Pathology, discussed the indication for treatment of each patient. Patients were selected for surgical resection if they were classified as Child-Pugh A or B, based on their predicted future liver remnant volumes, and according to their tumor stage, and Eastern Cooperative Oncology Group (ECOG) performance status.

Patients treated with TACE received either conventional Lipiodol-based TACE (cTACE), or TACE using drug-eluting beads (DEB-TACE). Treatment was performed in a standardized manner, as previously described [20,21]. After treatment, all patients received a control CT on the same day (cTACE) or the next day (DEB-TACE) to assess tumor staining and to exclude postinterventional complications. TACE was repeated every six weeks until viable tumor was no longer detected by CT or MRI or until any contraindications occurred [22]. SIRT-treated patients received two separate treatments with Yttrium-90-loaded resin spheres, one for the right and one for the left liver lobe, with four weeks between the two sessions. Systemic therapy was started with an initial dose of 400 mg b.i.d. sorafenib. This dose was reduced or the treatment was stopped in case of intolerable toxicity or clinical disease progression. CT and/or MRI were performed every three months to evaluate tumor response.

### Statistical analysis

Patient characteristics were described as mean or median and range for quantitative variables, and as absolute and relative frequency for categorical variables. OS was calculated as time from start of treatment to death. Censoring occurred at the end of the study or loss to follow-up. A descriptive analysis of survival was performed using Kaplan-Meier curves. Comparisons were based on the log-rank test.

As this study was not a randomized trial, it is highly likely that treatment decisions were influenced by PVTT sub-stage or other potentially prognosis-related factors. Thus, confounding is likely to be present. To adjust for possible confounders, we fitted a Cox regression model, with adjustment for PVTT extent, age, sex, Child-Pugh, ECOG, tumor size, and alpha-fetoprotein (AFP). We also applied propensity score weighting using the inverse probability of treatment weights in Cox regression models, to estimate both the average treatment effects (ATE) and the average treatment effects of the treated (ATT). Propensity scores were determined in a logistic regression model with therapy as a multinomial outcome and PVTT extent, age, sex, ECOG, tumor size, and AFP as covariates [23]. Patients with ECOG 2–3 or Child-Pugh C were excluded from all regression analyses because none of them underwent resection.

As this is a retrospective exploratory analysis, all *p*- values should be interpreted in a descriptive fashion. The term significant is used here to describe a *p*- value < 0.05.

Statistical analyses were performed using R 3.6.0 and SAS 9.4 [24,25].

## Results

### Patient characteristics at baseline

**Fig 2:**
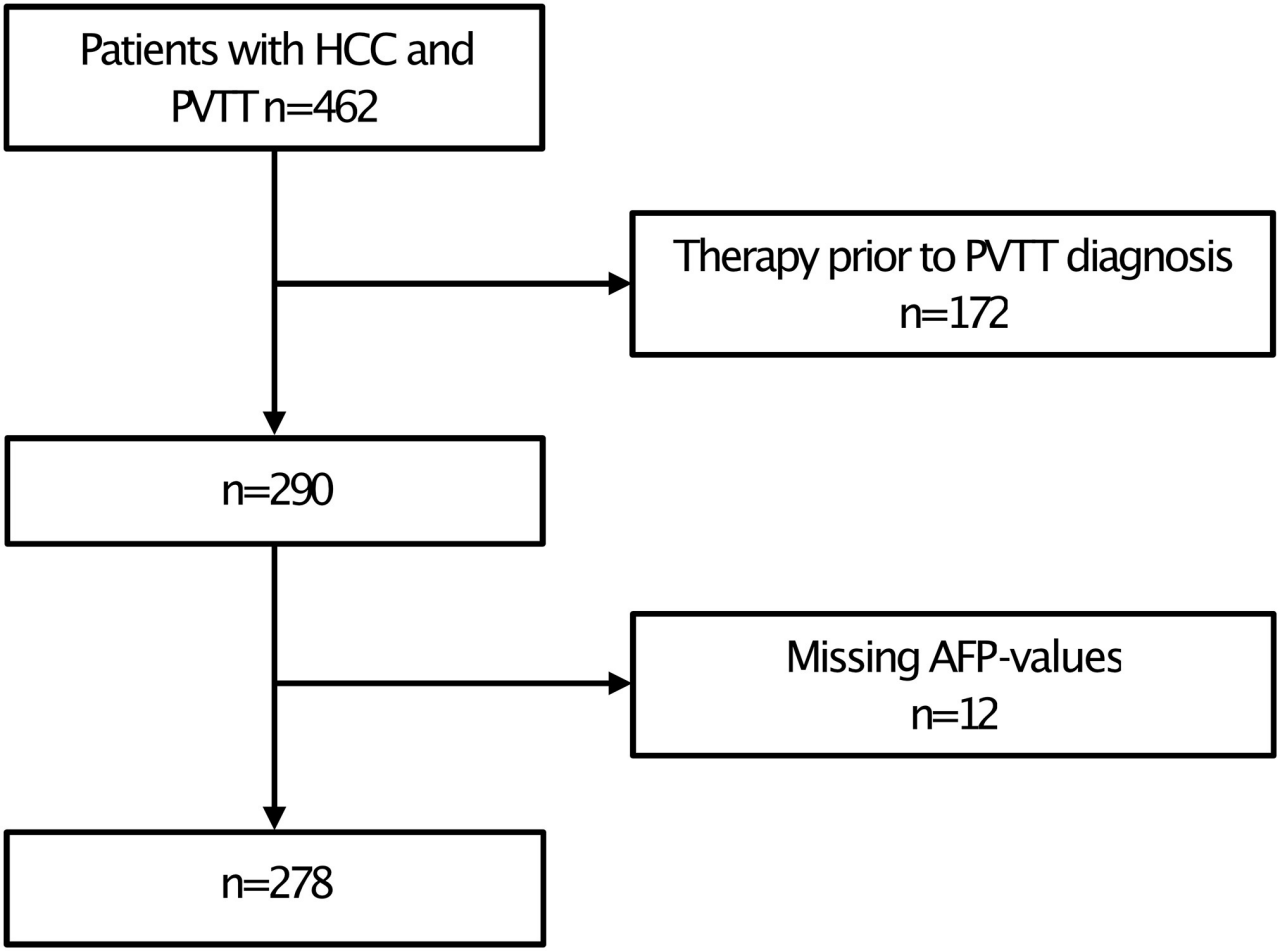
Flow diagram showing the reasons for patient exclusions, and the final number of patients included in the analysis. HCC, Hepatocellular carcinoma; PVTT, Portal vein tumor thrombosis; AFP, Alpha-fetoprotein.

A total of 462 patients matched the inclusion criteria, of whom 184 patients were excluded for the reasons indicated in Fig 2. Thus, 278 patients with HCC and PVTT were included in our analysis.

The follow-up time ranged from 0.1 to 104.2 months (mean 8.4 months). Table 1 presents baseline demographic data (including etiology, Child-Pugh stage, ECOG, AFP, tumor histology and tumor load), as well as PVTT classification and treatment.

**Table 1:** Patient characteristics at initial HCC diagnosis, PVTT classification, and treatment.

**Total number**	278
**Age in years, mean (range)**	65.6 (27–87)
**Gender, n (%)**	
Male	231 (83.1)
Female	47 (16.9)
**Etiology, n (%)**	
Alcoholic liver disease	114 (41.0)
Hepatitis C	48 (17.3)
Hepatitis B	35 (12.6)
NASH	16 (5.7)
Hemochromatosis	5 (1.8)
PBC/PSC	2 (0.7)
Unknown etiology	58 (20.9)
**Child-Pugh stage, n (%)**	
No liver cirrhosis	46 (16.5)
A	78 (28.1)
B	107 (38.5)
C	47 (16.9)
**ECOG, n (%)**	
0	108 (38.8)
1	142 (51.1)
2	23 (8.3)
3	5 (1.8)
**AFP in ng/mL, median (range)**	492 (1.4–629592)
≤200 ng/mL, n (%)	117 (42.1)
>200 ng/mL, n (%)	161 (57.9)
**Histological tumor type, n (%)**^a^	
Conventional HCC	230 (98.7)
Fibrolamellar HCC	3 (1.3)
**Growth pattern, n (%)**	
Diffuse growth pattern	104 (37.4)
Nodular growth pattern	174 (62.6)
**Diameter of the largest lesion, n (%)**^b^	
≤ 5 cm	51 (18.3)
> 5 cm	123 (44.2)
**Tumor number n (%)**^b^	
1	73 (41.9)
2	29 (16.7)
3	19 (10.9)
4	15 (8.6)
5	6 (3.4)
6	4 (2.3)
7	4 (2.3)
8	3 (1.7)
9	4 (2.3)
≥ 10	17 (9.8)
**Histological grading, n (%)**^a^^,^^c^	
G1	42 (18.0)
G2	106 (45.5)
G3	59 (25.3)
**PVTT Classification, n (%)**^d^	
Vp1	45 (16.2)
Vp2	54 (19.4)
Vp3	89 (32.0)
Vp4	90 (32.4)
**Therapy, n (%)**	
Resection	39 (14.0)
TACE/SIRT^e^	128 (46.0)
Sorafenib	57 (20.5)
BSC	54 (19.4)

NASH, Non-alcoholic steatohepatitis; PBC, Primary biliary cholangitis; PSC, Primary sclerosing cholangitis; ECOG, Eastern Cooperative Oncology Group; AFP, Alpha-fetoprotein; HCC, Hepatocellular carcinoma; G, Grading; PVTT, Portal vein tumor thrombosis; TACE, Transarterial chemoembolization; SIRT, Selective internal radiation therapy; BSC Best supportive care.

^a^ Histopathological proof was available for 233 patients (= 83.8%), in the remainder (n = 45; 16.2%) the HCC was diagnosed by a typical cross sectional imaging appearance of the tumor (according to EASL and AASLD guidelines).

^b^ Diameter of the largest lesion and tumor number is only available for nodular growth pattern.

^c^ In 26 (11.2%) patients with histopathological proof, the information about differentiation grade was missing.

^d^ Vp1 = segmental PV invasion; Vp2 = right anterior or posterior PV; Vp3 = right or left PV; and Vp4 = main trunk and/or contra-lateral portal vein branch to the primarily involved lobe.

^e^ 124 patients were treated with TACE, 4 patients were treated with SIRT.

### Survival of all patients

**Fig 3:**
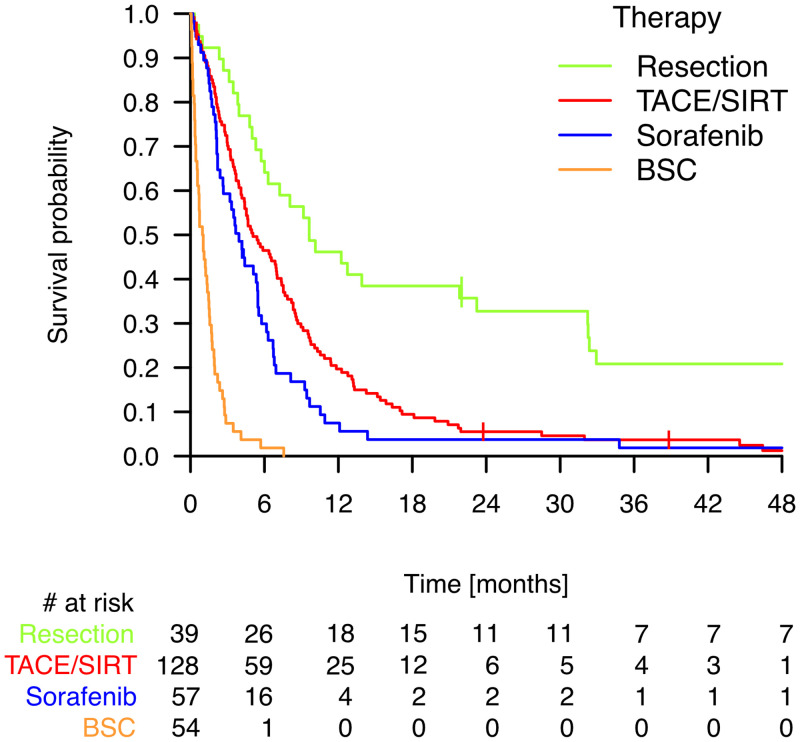
Kaplan-Meier curves comparing overall survival of patients undergoing different therapies. TACE, Transarterial chemoembolization; SIRT, Selective internal radiation therapy; BSC, Best supportive care.

The median OS in months was 9.63 (5.73–23.23) with resection, 5.07 (4.17–7.00) with TACE/SIRT, 3.93 (2.40–5.43) with sorafenib, and 1.00 (0.57–1.37) with BSC. The Kaplan-Meier curves (Fig 3) demonstrated a significantly longer OS of patients undergoing resection compared to treatment with sorafenib or BSC (*p* < 0.001). Furthermore, patients receiving TACE/SIRT had an only marginally better OS than patients treated with sorafenib (*p* = 0.059), but had a substantially better OS than patients treated with BSC (*p* < 0.001). Among all treatments, patients undergoing BSC had the worst OS (*p* < 0.001).

### Survival with different treatments according to PVTT extent

**Fig 4:**
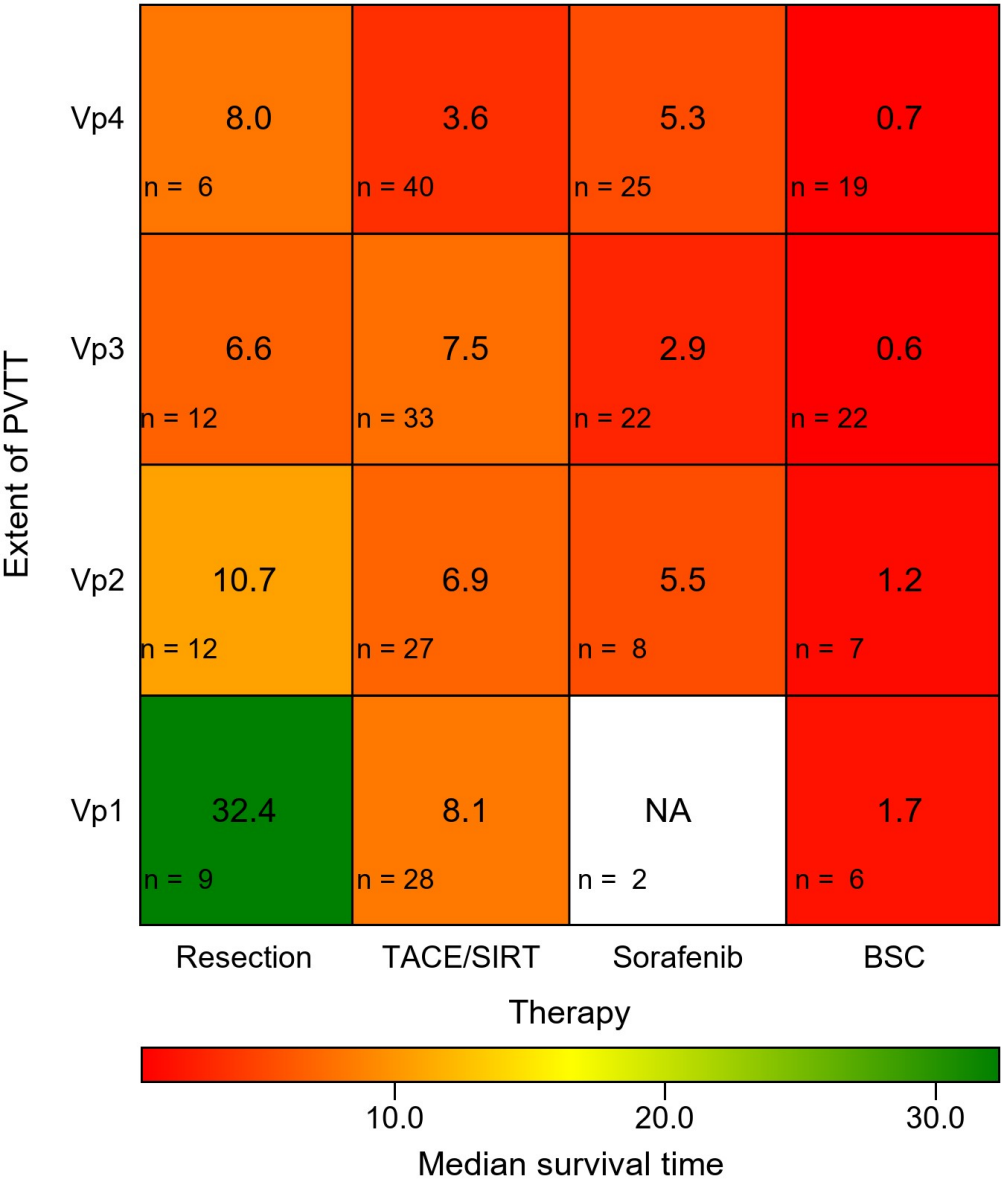
Median overall survival according to treatment modalities and PVTT stage (Vp1–Vp4). Middle text field: Median OS; lower left corner: Number of patients; NA: Not available because n = 2. PVTT, Portal vein tumor thrombosis; TACE, Transarterial chemoembolization; SIRT, Selective internal radiation therapy; BSC Best supportive care.

We further performed a subgroup analysis, comparing the different treatment modalities within each PVTT subgroup. Patients with segmental PVTT (Vp1) who underwent resection showed a considerably longer OS (32.4 months) compared to all other treatments (all *p* values < 0.01). We observed no significant difference between the TACE/SIRT and sorafenib groups, except within the PVTT substage Vp3 (*p* = 0.026). In general, patients treated with BSC presented the lowest OS across all PVTT stages. Fig 4 shows all combinations of PVTT stages and types of treatment.

S1 Fig shows the corresponding Kaplan Meier curves for the different portal vein tumor thrombosis cohorts.

### Adjustment for risk factors

To adjust for possible confounders and to investigate how our results depended on possible influencing factors, we performed Cox regression and propensity score analyses.

#### Cox regression analysis with adjustment for risk factors

Cox regression analysis was performed with adjustment for established risk factors, including PVTT extent, age, sex, Child-Pugh, ECOG, tumor size, and AFP. With adjustment, resection still yielded the best OS compared to all other therapies: resection vs. TACE/SIRT, HR 0.52, 95% CI [0.32, 0.83]; resection vs. sorafenib, HR 0.45, 95% CI [0.26, 0.77]; and resection vs. BSC, HR 0.12, 95% CI [0.06, 0.23]. TACE/SIRT showed little survival benefit over sorafenib: HR 0.87, 95% CI [0.60, 1.26]. BSC was associated with the worst survival: resection vs. BSC, HR 0.12, 95% CI [0.06, 0.23]; TACE/SIRT vs. BSC, HR 0.22, 95% CI [0.13, 0.39]; and sorafenib vs. BSC, HR 0.26, 95% CI [0.15, 0.45]. In this analysis we also observed a reduced risk in females (HR 0.62, 95%CI [0.41, 0.92]) and an increased risk for patients with ECOG 1 (compared to ECOG 0) with HR 1.41, 95%CI [1.05, 1.90] and for patients with Vp4 compared to Vp1 (HR 1.75, 95%CI [1.08, 2.81]). The other adjustment variables (age, Child Pugh, tumor size and AFP) did not exhibit strong association with survival.

#### Cox regression analysis with adjustment for risk factors and additional propensity score analysis (ATE analysis)

We next performed a Cox regression analysis with adjustment for all risk factors (as above) as well as additional propensity score weighting (doubly robust analysis). This analysis essentially confirmed the results of the first analysis. Again, OS was better with resection compared to all other therapies: resection vs. TACE/SIRT, HR 0.58, 95% CI [0.36, 0.92]; resection vs. sorafenib, HR 0.55, 95% CI [0.34, 0.89]; and resection vs. BSC, HR 0.09, 95% CI [0.04, 0.19]. Little difference was observed between TACE/SIRT vs. sorafenib: HR 0.95, 95% CI [0.64, 1.41]. BSC was associated with the worst survival: resection vs. BSC, HR 0.09, 95% CI [0.04, 0.19]; TACE/SIRT vs. BSC, HR 0.16, 95% CI [0.08, 0.30]; and sorafenib vs. BSC, HR 0.16, 95% CI [0.08, 0.32].

#### ATT analysis

Finally, we performed an ATT analysis to investigate what would have happened if patients treated with TACE/SIRT had undergone resection instead. ATT analysis was performed looking at a Cox regression model with PVTT, as well as at therapies and at their interactions. The results showed that patients with segmental PVTT (Vp1) might have benefited from resection (HR 0.43, 95% CI [0.19, 0.95]). For all other PVTT subgroups (Vp2–Vp4), we found no evidence that they would have significantly benefited from surgery. Among patients with Vp1, those who were actually resected showed an HR of 0.26 (95% CI [0.12, 0.58]) when comparing resection to TACE/SIRT.

We also performed another ATT analysis to investigate what would have happened if patients treated with sorafenib had instead undergone resection. There were too few patients with segmental PVTT (Vp1) who received sorafenib to assess their potential benefit from resection. Among all other PVTT subgroups (Vp2–Vp4), we found no significant evidence that they would have benefited from surgery.

## Discussion

In this study, we aimed to identify patients with HCC and PVTT who might benefit from individualized therapeutic approaches other than systemic therapy, particularly considering the extent of PVTT. We demonstrated that survival among patients with advanced HCC considerably varied according to different therapies and to the extent of PVTT. Notably, patients with segmental PVTT (Vp1) who underwent resection had a significantly longer OS compared to any other treatment group. This prompts the hypothesis that for well-selected patients with minor segmental PVTT, hepatic resection might provide better outcomes than TACE/SIRT or sorafenib, even within this Western patient cohort.

In various Asian guidelines, PVTT is not a strict contraindication for potentially curative treatment options like resection; instead, more aggressive management recommendations have been established for such cases [26–29]. This stands in contrast to the Western world, where, following the BCLC recommendations, the AASLD and EASL guidelines consider systemic palliative therapy as the only recommended standard of care [5–8]. However, in clinical practice, patients receive a wide variety of therapies not only in Asia, but also in the Western world, including resection, TACE, and SIRT [11,12]. Since sorafenib therapy provides only a modest OS benefit, it is quite common that patients are offered other treatment options in the hope for a better outcome. This likely happens at least partly due to underestimation of the negative prognostic impact of a minor PVTT. Furthermore, PVTT is frequently missed in imaging during daily clinical practice [30].

Our present study is the first analysis of a Western patient cohort to demonstrate that selected Caucasian patients may also benefit from resection in case of Vp1-PVTT. However, this was not the case for more advanced stages of PVTT (Vp2–Vp4). In Asian liver centers, hepatic resection with or without tumor thrombectomy is currently a widespread practice for selected patients with HCC and PVTT, even when PVTT is more extensive than Vp1 [13,31,32]. Kokudo et al. found that in patients with PVTT stages Vp1–Vp3, resection conferred a survival benefit compared to patients who were not resected (treated with TACE, chemotherapy or hepatic arterial infusion chemotherapy, ablation therapy, best supportive care, and other treatments); however, patients with Vp4 did not benefit from resection in terms of OS [13]. In the meta-analysis by Liang et al., among patients with Vp1–3, the hazard ratios for the 1-, 3-, and 5-year OS rates were also in favor of the resection subgroup compared to the non-resection subgroup undergoing various therapies (TACE, sorafenib, TACE combined with sorafenib, TACE combined with radiotherapy) [32]. Again, for patients with Vp4, they found no significant difference between the resection and non-resection subgroups [32]. Overall, several Asian studies indicate that surgery can yield survival benefits and enhanced quality of life for selected patients (Vp1–Vp3), but should be cautiously considered for patients with PVTT invading the main trunk of the portal vein (Vp4), as this may increase the risk of postoperative complications and liver failure [13,31,32]. Our present findings are basically in line with these prior results, with the exception that in our Western patient cohort, resection led to a survival benefit only in patients with Vp1.

In cases of unresectable HCC, TACE plays an established role in treatment [33–35]. However, TACE is theoretically contraindicated in the presence of PVTT because of the potential risk of liver necrosis due to post-TACE ischemia. In a meta-analysis, Zhang et al. demonstrated that hepatectomy was superior to TACE in patients with peripheral PVTT, but did not find a significant survival difference between hepatectomy and TACE among patients with main trunk PVTT [14]. Furthermore, another group performed a meta-analysis and found a survival benefit of resection compared to TACE among patients with Vp1–3, but not Vp4 [36].

Compared to BSC, TACE has yielded a better OS in patients with any extent of PVTT in several Asian trials [37–39]. However, another research group found that TACE achieved an OS benefit for patients with Vp1–3, but not Vp4 [40]. Overall, these previous findings are in accordance with our present results in which patients undergoing BSC exhibited the lowest OS across all PVTT substages.

Regarding the use of sorafenib as standard therapy, our present results are consistent with the findings of the phase III Sorafenib Hepatocellular Carcinoma Assessment Randomized Protocol (SHARP) study (OS of 10.7 months in the sorafenib group vs. 7.9 months in the placebo group, *p* < 0.001) and the Asia-Pacific trial (OS of 6.5 months in the sorafenib group vs. 4.2 months in the placebo group, *p* < 0.05) [9,10]. Notably, a subgroup analysis of the SHARP trial revealed that among patients with macrovascular invasion, sorafenib therapy yielded a longer median OS (8.1 months vs. 4.9 months) and time to progression (4.1 months vs. 2.7 months) compared to placebo [41]. However, neither trials did take into account PVTT extent [9,10].

The present study has several limitations. The most important limitation was the lack of randomization. Treatment decisions were determined in clinical reality, accounting for numerous factors such as patient characteristics or tumor characteristics. This poses the risk of a potential selection bias in our population, as patients in a better clinical condition (for example patients with ECOG 0) are more likely to undergo more aggressive therapies, like TACE/SIRT or even resection. In the absence of data from randomized controlled clinical trials, we performed analyses with adjustment for possible confounders and used propensity score weighting (doubly robust analysis), thus mimicking a randomized clinical trial and compensating for differences in the covariates between patients receiving different treatments. However, even with this approach, we may not have completely avoided the biases arising from the retrospective study design. Another limitation was the relatively small number of patients in the PVTT subgroups of each treatment, which might have led to underpower. However, most studies in the literature do not differentiate between different PVTT substages, and thus do not account for possible differences in patients’ prognosis. Notably, CT and MRI scanners and protocols have been continuously improved, such that it is now easier to diagnose small PVTT. Both investigators had a high level of expertise in HCC imaging, and paid particular attention to PVTT detection. Additionally, they analyzed the images retrospectively, considering the course of disease. Therefore, considerably more PVTTs were detected in our present study than in clinical practice. It should be noted that our patient cohort may have shown a tendency towards more advanced tumors, as our hospital is a specialized tertiary care liver center. Finally, due to differences in underlying liver diseases, our results may not be applicable to patients with HCC and PVTT in other countries. For example, Asian studies report a much higher proportion of patients with chronic hepatitis B—a population that often develops HCC in a non-cirrhotic liver [2,42]. Moreover, Asian patients generally have fewer comorbidities. Thus, it may not be possible to transfer our results to Asian patients.

In this study, all patients undergoing systemic therapy received sorafenib. However, in the last two years, several new agents have become available for systemic therapy. Lenvatinib is proven to be a non-inferior first-line treatment option compared to sorafenib; however, that study excluded patients with main portal trunk PVTT [43]. Regorafenib [44] and Cabozantinib [45] have been demonstrated to be effective as second-line option in selected patients with sorafenib-resistant HCC. The immunotherapeutic drug Pembrolizumab [46] (NCT02702414) is already approved by the Food and Drug Administration (FDA) as second-line treatment. Nivolumab has been approved by the FDA, but did not reach the primary endpoint in Check-Mate-459 (NCT02576509). The combination of Atezolizumab and Bevacizumab has shown promising results in the IMBRAVE 150 trial [47]. However, sorafenib is still the mainstay of systemic therapies. Further studies investigating these new agents are warranted.

## Conclusions

This study was the first to compare different treatments in different PVTT substages among Western patients. Our results showed that hepatic resection was beneficial for patients with segmental PVTT (Vp1), and provided better outcomes than TACE/SIRT or sorafenib. These findings suggest that for well-selected patients with minor PVTT, resection may be an appropriate alternative therapeutic option when deemed oncologically reasonable.

## Supporting information

S1 Checklist STROBE statement—Checklist of items that should be included in reports of observational studies. (DOC)

S1 Fig Kaplan Meier curves for the different portal vein thrombosis cohorts. A = Vp1, B = Vp2, C = Vp3, D = Vp4. TACE, Transarterial chemoembolization; SIRT, Selective internal radiation therapy; BSC, Best supportive care.
(TIFF)
