# Supplementary material for: The impact of portal vein tumor thrombosis on survival in patients with hepatocellular carcinoma treated with different therapies: A cohort study
Source: PLoS One. 2021 May 7;16(5):e0249426. doi: 10.1371/journal.pone.0249426 (PMC8104403; doi:10.1371/journal.pone.0249426)
Supplement: S1 Checklist — (DOC) [file pone.0249426.s001.doc]

STROBE Statement—checklist of items that should be included in reports of observational studies

|  | Item No | Recommendation | |
| --- | --- | --- | --- |
| **Title and abstract** | 1 | (*a*) Indicate the study’s design with a commonly used term in the title or the abstract  **p.1, l.3** | |
|  |  | (*b*) Provide in the abstract an informative and balanced summary of what was done and what was found  **p.2-3, l.34-51** | |
| Introduction | | | |
| Background/rationale | 2 | Explain the scientific background and rationale for the investigation being reported  **p.4, l.56-75** | |
| Objectives | 3 | State specific objectives, including any prespecified hypotheses  **p.4, l.74-75** | |
| Methods | | | |
| Study design | 4 | Present key elements of study design early in the paper  **p.5, l.80** | |
| Setting | 5 | Describe the setting, locations, and relevant dates, including periods of recruitment, exposure, follow-up, and data collection  **p.5, l.85-99** | |
| Participants | 6 | (*a*) *Cohort study*—Give the eligibility criteria, and the sources and methods of selection of participants. Describe methods of follow-up  **p.5, l.95-99** | |
|  |  | (*b*) *Cohort study*—For matched studies, give matching criteria and number of exposed and unexposed  **N/A** | |
| Variables | 7 | Clearly define all outcomes, exposures, predictors, potential confounders, and effect modifiers. Give diagnostic criteria, if applicable  **p.6, l.111-120; p.7, l.136-146; p.7, l.150-151; p.8, l.154-164** | |
| Data sources/ measurement | 8 | For each variable of interest, give sources of data and details of methods of assessment (measurement). Describe comparability of assessment methods if there is more than one group  **p.5, l.80; p.5, l.85-94; p.6-7, l.104-146; p.7, l.150-151** | |
| Bias | 9 | Describe any efforts to address potential sources of bias  **p.8, l.154-164** | |
| Study size | 10 | Explain how the study size was arrived at  **p.8, l.171-173 and Figure 2** | |
| Quantitative variables | 11 | Explain how quantitative variables were handled in the analyses. If applicable, describe which groupings were chosen and why  **p.7, l.149-150** | |
| Statistical methods | 12 | (*a*) Describe all statistical methods, including those used to control for confounding  **p.7-8, l.149-167** | |
|  |  | (*b*) Describe any methods used to examine subgroups and interactions  **p.7-8, l.149-167** | |
|  |  | (*c*) Explain how missing data were addressed  **p.8, l.162-164 and Figure 2** | |
|  |  | (*d*) *Cohort study*—If applicable, explain how loss to follow-up was addressed  **p.7, l.151-152** | |
|  |  | (*e*) Describe any sensitivity analyses  **p.8, l.165-166** | |
| Results | | | |
| Participants | 13 | (a) Report numbers of individuals at each stage of study—eg numbers potentially eligible, examined for eligibility, confirmed eligible, included in the study, completing follow-up, and analysed  **p.8, l.171-173 and Fig.2-4** | |
|  |  | (b) Give reasons for non-participation at each stage  **Fig.2** | |
|  |  | (c) Consider use of a flow diagram  **Fig.2** | |
| Descriptive data | 14 | (a) Give characteristics of study participants (eg demographic, clinical, social) and information on exposures and potential confounders  **p.9-10, Table 1** | |
|  |  | (b) Indicate number of participants with missing data for each variable of interest  **Fig.2** | |
|  |  | (c) *Cohort study*—Summarise follow-up time (eg, average and total amount)  **p.9, l.178** | |
| Outcome data | 15 | *Cohort study*—Report numbers of outcome events or summary measures over time  **p.11,** **l.197-203; p.11. l.210-216; Fig.3 and 4, Fig. S1** | |
| Main results | 16 | (*a*) Give unadjusted estimates and, if applicable, confounder-adjusted estimates and their precision (eg, 95% confidence interval). Make clear which confounders were adjusted for and why they were included  **p.12-13, l.226-254** | |
|  |  | (*b*) Report category boundaries when continuous variables were categorized  **Table 1** | |
|  |  | (*c*) If relevant, consider translating estimates of relative risk into absolute risk for a meaningful time period  **N/A** | |
| Other analyses | 17 | Report other analyses done—eg analyses of subgroups and interactions, and sensitivity analyses  **p.13, l.257-269** | |
| Discussion | | | |
| Key results | 18 | Summarise key results with reference to study objectives  **p.14, l.271-278; p.17, l.369-373** | |
| Limitations | 19 | Discuss limitations of the study, taking into account sources of potential bias or imprecision. Discuss both direction and magnitude of any potential bias **p.16-17, l.331-366** | |
| Interpretation | 20 | Give a cautious overall interpretation of results considering objectives, limitations, multiplicity of analyses, results from similar studies, and other relevant evidence  **p.14-17, l.271-373** | |
| Generalisability | 21 | Discuss the generalisability (external validity) of the study results  **p.15, l.318-328** | |
| Other information | | | |
| Funding | 22 | Give the source of funding and the role of the funders for the present study and, if applicable, for the original study on which the present article is based  **N/A** |  |
