# Supplementary figures and images for: The impact of portal vein tumor thrombosis on survival in patients with hepatocellular carcinoma treated with different therapies: A cohort study
Source: PLoS One. 2021 May 7;16(5):e0249426. doi: 10.1371/journal.pone.0249426 (PMC8104403; doi:10.1371/journal.pone.0249426)

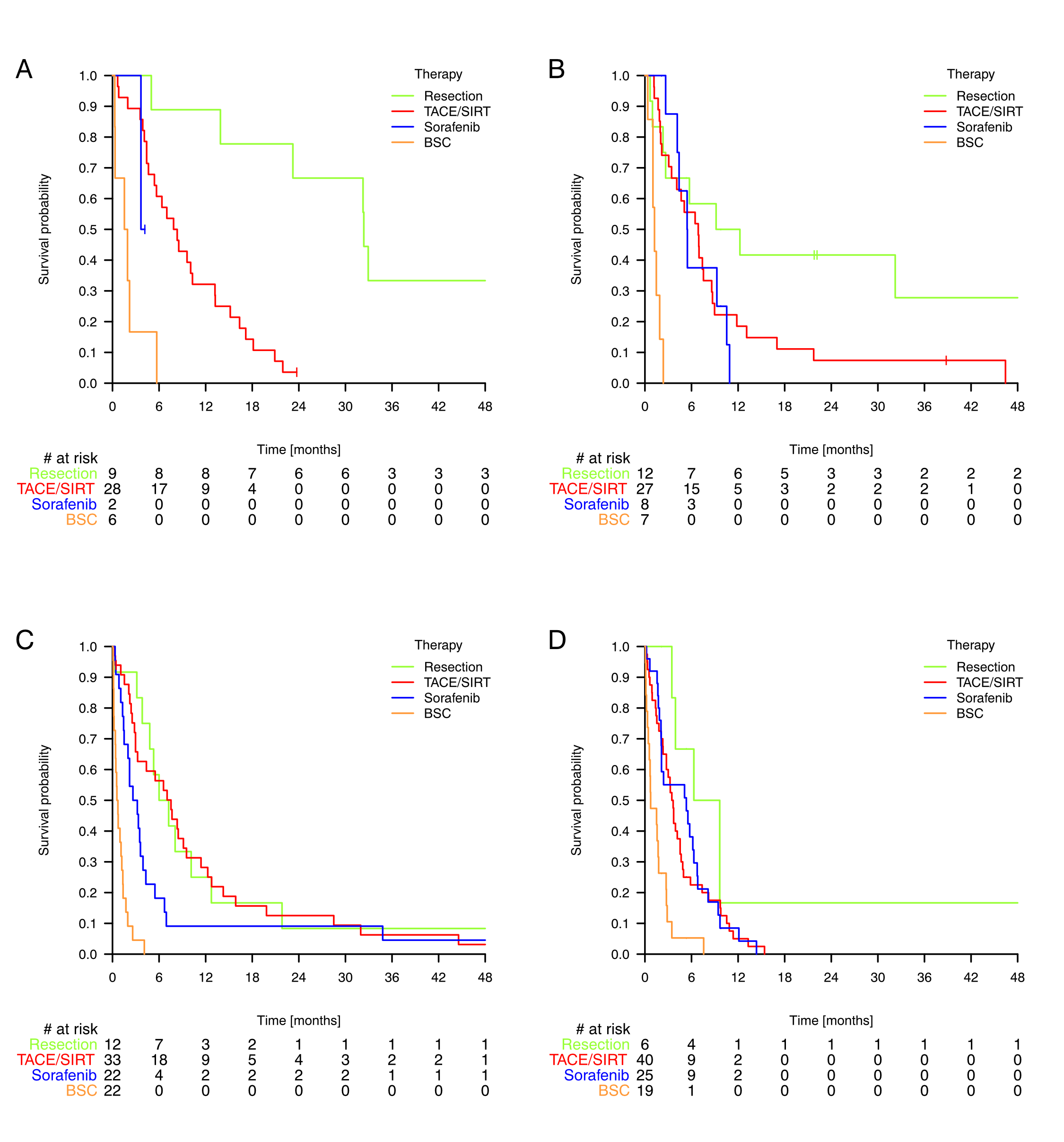

Supplement: S1 Fig — A = Vp1, B = Vp2, C = Vp3, D = Vp4. TACE, Transarterial chemoembolization; SIRT, Selective internal radiation therapy; BSC, Best supportive care. (TIFF) [file pone.0249426.s002.tiff]
